# Urgent Care: the evolution of a revolution

**DOI:** 10.1186/2045-4015-2-39

**Published:** 2013-10-23

**Authors:** Lee A Resnick

**Affiliations:** 1 JUCM, The Journal of Urgent Care Medicine, 120 N Central Avenue, Ramsey, NJ, 07446, USA

## Abstract

The rapid and global growth of urgent care centers has had a revolutionary, though poorly understood, impact on the health care delivery system. The consumer-driven care model inherent in urgent care and other so-called convenience care models is permeating into more conventional health care models. In addition, physician and hospital payment models are evolving, especially in the United States and are creating new market forces that will impact the organization and importance of integrated health care networks in the future. Together, these transformative changes are creating evolutionary pressures on traditional urgent care. Lessons learned from both the Israeli and American experience can be especially helpful for drafting a successful urgent care model for the future. This is a commentary on http://www.ijhpr.org/content/2/1/38/.

## Commentary

In her comprehensive review, “Community-Based Urgent Care in Israel and Worldwide” [1], Dr. Deena Zimmerman explores the market and health care delivery trends behind the rapid explosion of urgent care centers globally and the impact such trends have had on her native Israel. Dr Zimmerman provides a substantial review of the state of urgent care in the United States, where there has been rapid growth for about 20 years now, reaching what many now say is over 10,000 urgent care centers nationwide. Despite the size of the industry, there has been little research to date, and there are many gaps in our understanding of the current and potential impact of this hybrid model of emergency care and primary care. Dr Zimmerman astutely identifies the different influences driving the early growth of urgent care in the United States versus Israel, with entrepreneurial physicians leading the former and insurers behind the latter. It is here, at the convergence of these two unique paths, where I find the most fascinating and transformative aspects of the evolution of this discipline.

At the heart of the Israeli model is “managed care” and its emphasis on finding solutions that reduce cost and improve efficiency while reducing unnecessary utilization. The nation quickly discovered the convenience virtues of urgent care and patients consistently rated their experiences higher than at other health system access points, including their own personal physicians. Thus the convergence of managed care and consumer-driven care has led to a rapid growth of such centers in Israel. Many other countries, with similar health care delivery models, have seen similar trends.

So what about the United States, where “cowboy” physicians started it all? Well, the urgent care marketplace is still a dynamic one. And while some see a surge of private equity investors, with their acquisitions and rapid scalability, as the most important trend in the industry, I propose that the more macroeconomic influences like national health care reform are far more telling. Regardless of what political leftovers will remain of “Obamacare,” there is one certainty: Payment reform is going to happen. On one side of the political spectrum, you have calls for a single-payer model, with the rationing and price pressures that follow. On the other end is a conservative movement bent on reducing federal spending on health care, leaving individuals and employers with little choice but to reduce health care costs in the name of sustainable premiums. In the middle are the insurance companies, looking to capitalize on all this penny pinching by offering health plans that reward smart consumer choices and reimburse doctors and hospitals through population health payment models. All told, we are watching the managed care model of the 1980s get flipped on its head. In that original model, insurance companies literally *imposed* closed networks and rationed care on the consumer and on physicians and hospitals. In this modern iteration, they have cleverly created reimbursement models that drive the same behavior *without* imposing it. Payments determined by “population health” and delivery efficiencies have given rise to market forces that encourage utilization and cost management all on their own. No top-down mandates or so-called “socialist” ideals necessary. It is this trend, this tactical reimbursement re-structuring, that will dictate the future of health care in America and, consequently, the role of urgent care in it.

Urgent care in America was born from a revolutionary vision of health care and led by physician innovators who recognized weakness and gaps in health care delivery. These shortcomings became opportunities for entrepreneurs to offer a superior health care product, and quickly became a haven for physicians fed up with the status quo of conventional medicine. The burdens of traditional health care were largely absent in urgent care and physicians were eager to practice medicine free of many of the administrative hassles and paperwork drowning our primary care colleagues. Urgent cares were, for the most part, welcomed with open arms by the communities they served and the insurance companies were eager to contract with them in an effort to support an emergency department diversion strategy. For the last 15 years or so, urgent cares have flourished under these favorable economic and professional conditions [2].

But while the excitement behind all revolutions eventually fades, the need for evolution endures. Likewise, many innovative ideas succeed in the short term only to fade for lack of vision and ability to change with the fickle market and consumer demands. Urgent care exists in this evolutionary moment. The emphasis on population health and integrated networks, as previously described, leaves little room for an independent urgent care practice. Employers and health systems will be forced to create closed, integrated networks that direct care through efficient and controlled pathways. In response, health systems are frantically moving into the urgent care business, seeing it as one of their most important strategies for preventing leakage, capturing new market share and managing costs. Largely ignored by health systems for the last 15 years, urgent care is now a mandate. The urgency has created pressure to grow de novo networks quickly or to create formal partnerships or outright acquisitions. Given these influences, the urgent care landscape will change quickly. Urgent care centers that refuse to participate in the newly created networks could quickly find themselves squeezed out of the game, with shrinking volumes and influence.

In the end, the American urgent care model will look much more like the Israeli model, integrated and aligned with health systems and insurance companies. We still have tremendous opportunity to play an important and profitable role in health care delivery, but an isolationist or revolutionary stubbornness will fail those drunk on the easy profits of the last 15 years. The message to American urgent cares is clear: Evolve and integrate or die.

## Competing interests

The author declares that he has no competing interests.

## Authors’ information

Dr Resnick is Editor-In-Chief of JUCM, The Journal of Urgent Care Medicine. He also serves as Chief Medical Officer of WellStreet Urgent Care in Atlanta, Georgia, and is an Assistant Clinical Professor with the Department of Family Medicine at Case Western Reserve University in Cleveland, Ohio. He formerly served as President of the Urgent Care Association of America.

## Commentary on

Zimmerman DR: Community-based urgent care in Israel and worldwide. Isr J Health Policy Res 2013, 2:38.
